# Repeated restraint stress-induced increase in post-surgical somatosensory hypersensitivity and affective responding is mediated by β-adrenergic receptor activation and spinal NLRP3-IL1β signalling in male rats

**DOI:** 10.1038/s41386-025-02305-x

**Published:** 2026-01-09

**Authors:** Ariadni Bella, Khaled Abdallah, Daniela Rodrigues-Amorim, Alba M. Diego, Charles Decraene, Pierre Hener, Chiara Di Marino, David P. Finn, Ipek Yalcin, Michelle Roche

**Affiliations:** 1 Physiology, School of Pharmacy and Medical Sciences, College of Medicine, Nursing and Health Sciences, University of Galway, Galway, Ireland; 2 Galway Neuroscience Centre, University of Galway, Galway, Ireland; 3 Centre for Pain Research, University of Galway, Galway, Ireland; 4 Centre National De la Recherche Scientifique, Institut des Neurosciences Cellulaires et Intégratives, Université de Strasbourg, Strasbourg, France; 5 Pharmacology and Therapeutics, School of Pharmacy and Medical Sciences, College of Medicine, Nursing and Health Sciences, University of Galway, Galway, Ireland

**Keywords:** Chronic pain, Stress and resilience, Neuroimmunology

## Abstract

Pre-surgical stress is a well-recognised risk factor for persistent post-surgical pain, and while the precise underlying neurobiological mechanisms remain unknown, neuro-immune interactions are believed to play a pivotal role. Here, we investigated the effect of repeated restraint stress (RRS) on post-surgical somatosensory hypersensitivity and affective responding in male rats and examined underlying mechanisms. We showed that RRS induced behavioural despair, reduced body weight gain and elevated faecal corticosterone levels in male Sprague-Dawley rats. Following paw incision surgery, animals pre-exposed to RRS exhibited exacerbated mechanical and heat hypersensitivity, pain-related aversion, and anxiety-like behaviour compared to non-stress counterparts. RNAseq analysis revealed alterations in expression of glial markers and inflammasome pathways in the dorsal horn of the spinal cord in the RRS + paw incision group, compared to paw incision alone, data further confirmed by immunohistochemistry and RT-qPCR analysis. Intrathecal administration of Interleukin-1 receptor antagonist (IL-1Ra) or MCC950 (an NLRP3 inhibitor) attenuated the RRS-induced increase in pain-related aversion and mechanical hypersensitivity post-surgery. Chronic administration of the β-adrenergic receptor antagonist propranolol, but not the glucocorticoid receptor antagonist RU486, attenuated the RRS-induced exacerbation of mechanical hypersensitivity, pain-related aversion and anxiety-like behaviour post-surgery. These findings suggest that RRS exacerbates and prolongs post-surgical somatosensory and affective pain responding via β-adrenergic receptor activation and increased spinal microglial NLRP3-IL-1β signalling. These data provide further insight into the mechanisms by which chronic stress and mood disorders exacerbate and prolong post-surgical pain.

## Introduction

Each year, over 300 million people undergo surgery worldwide [1]. While acute post-surgical pain is an expected and manageable outcome, 10–50% of patients develop prolonged and persistent post-surgical pain (PPP) [2]. This transition from acute to persistent pain presents a significant clinical challenge, often complicated by psychological factors and pre-existing mood disorders, such as depression, which is linked to greater pain intensity and delayed recovery [3–6]. Despite growing interest in the link between emotional dysregulation and post-surgical pain, our understanding of the mechanisms underlying these interactions remains limited, highlighting the need for further research to improve patient outcomes.

Animal models, such as the hind paw incision model, have provided critical insights into the mechanisms underlying post-surgical pain. In this model, animals exhibit mechanical and heat hypersensitivity [7], as well as anxiety-like behaviour [8, 9], which typically resolve within five to seven days. However, exposure to stress prior to surgery can result in more intense and prolonged post-surgical sensory hypersensitivity. Acute stress in the form of a single prolonged stress (SPS) episode resulted in prolonged mechanical hypersensitivity for up to 35 days post-surgery [10–13]. Similarly, pre-operative REM sleep disturbance (RSD) [14–16] or repeated immobilization stress for 3 days [17] increased mechanical and heat hypersensitivity in male rats for up to 9 days post paw-incision surgery. Chronic stress paradigms such as repeated restraint/immobilisation (RRS) or repeated defeat stress (RDS) increased the magnitude and duration of post-surgical mechanical hypersensitivity for between 9 and 24 days [18–22]. However, the effect of stress on other pain-related outcomes such as heat hypersensitivity, aversive behaviour and affective responding is unknown.

Several mechanisms may underlie the effects of stress on the prolongation of post-surgical pain [for review see [23]]. For example, SPS has been found to increase proinflammatory cytokine levels and lead to spinal astrocyte activation for up to 28 days post-surgery [10], while inhibition of astrocyte activation was able to prevent the exacerbation of mechanical hypersensitivity post-surgery [13]. Furthermore, minocycline (a multi-action antibiotic, which can inhibit microglia reactivity) [24], an alpha7 nicotinic acetylcholine receptor (α7 nAChR) antagonist [11] or GSK650394 (a serum- and glucocorticoid-regulated kinase 1 (SGK1) inhibitor) [13], attenuated SPS-induced potentiation of mechanical hypersensitivity and spinal neuroinflammation. Few studies have examined mechanisms underlying the effects of chronic stress on post-surgical pain. Increased NLRP3-IL-1β signalling in the basolateral amygdala (BLA) shown to mediate the prolongation of post-surgical mechanical hypersensitivity following RRS [22], however, the role of spinal neuroimmune mechanisms is unknown.

Recent perspectives highlight that the concept of “microglial activation” is overly simplistic, as microglia can adopt a wide range of context-dependent functional states rather than switching simply between “resting” and “activated” states [25, 26]. Such shifts in microglial morphology, gene expression, and cytokine signalling reflect adaptive responses to physiological or environmental stimuli, as well as potential indicators of neuroinflammatory processes. Within this framework, stress can prime microglia and alter their phenotype, creating a neuroimmune environment more susceptible to exaggerated responses upon subsequent challenges [27–33]. Repeated social defeat stress–induced allodynia has been associated with increased microglial reactivity within the spinal dorsal horn, along with elevated levels of pro-inflammatory mediators [34]. Activation of the two key stress pathways, the hypothalamic–pituitary–adrenal axis (HPA axis) and the sympathetic nervous system (SNS), has been shown to increase microglia reactivity, leading to increased production of IL-1β and TNF-α [33, 35–37]. For example, Feng and colleagues demonstrated that RRS increases the expression of *Iba1*, *Il-1β* and *Il-18* in hippocampal microglia and induces depressive-like behaviour, effects mediated by activation of the glucocorticoid receptor-NF-κB-NLRP3 signalling pathway [35]. In addition, antagonism of the glucocorticoid receptor has been shown to prevent RRS-induced neuroimmune alterations in the spinal cord [38]. Inhibition of β-adrenergic activation has been shown to block repeated social defeat-induced anxiety-like behaviour and enhanced neuroimmune mediators at a supraspinal level [33]. Thus, glucocorticoid and β-adrenergic receptors play a pivotal role in mediating chronic stress-induced neuroimmune modulation and may underlie the effects of chronic stress on post-surgical pain responding.

We hypothesised that chronic stress would result in glucocorticoid and/or β-adrenergic receptor activation and spinal microglial priming, resulting in enhancement of NLRP3-IL-1β signalling, prolongation of mechanical and heat hypersensitivity and exacerbation of affective pain responding post-surgery. To test this hypothesis, we used RRS combined with the hind paw incision model of post-surgical pain to develop a model of prolonged post-surgical hypersensitivity and affective responding and used a combination of molecular and pharmacological approaches to uncover the mechanisms involved.

## Methods

### Animals

A total of 288 adult male Sprague-Dawley rats (170–220 g; Janvier, France) were used for the experiments. Male rats were selected for this study based on our pilot data demonstrating that, although the same RRS protocol prolonged post-surgical pain behaviours in both male and female rats, it did not lead to increased spinal *Iba1*, *Nlrp3* or *Il-1β* expression in females, suggesting the involvement of sex-specific mechanisms (data not shown; mechanisms in females are the focus of a separate study). Animals were housed in groups of two or three in type IV cages (55x33x20cm) under a 12:12 h light–dark cycle (lights on at 8am) and maintained at controlled conditions (temperature 22 ± 2 °C, humidity 45–55%), with food and water *ad libitum*. All experiments were carried out during the light phase. The experimental procedures were approved by the Animal Care and Research Ethics Committee, National University of Ireland Galway and carried out under license from the Health Products Regulatory Authority in the Republic of Ireland and approval by the regional ethical committee of Strasbourg (CREMEAS, APAFIS 43704) in accordance with EU Directive 2010/63 and the ARRIVE guidelines.

### Experimental design

A graphical representation of the experimental design is provided in Supplementary Fig. 1 (S1). For all experiments, animals were assigned randomly to experimental groups.

#### Establishment of the chronic stress-induced potentiation of post-surgical somatosensory hypersensitivity model

Rats were exposed to RRS or no RRS (handling) for 6 h/day over 21 days. Shorter RRS protocols (6 h/day for 3 days and 2.5 h/day for 14 days) were also tested but did not alter post-surgical hypersensitivity (see Supplementary Figs. S3 and S4). Body weight gain, mechanical and heat sensitivity were assessed throughout the RRS procedure, and the forced swim test (FST) was conducted following the last RRS session. One day following the end of RRS, animals underwent paw incision or sham surgery. Mechanical (von Frey) and heat (Hargreaves’ test) sensitivity were assessed for up to 3 weeks following paw incision or sham surgery. Power analysis was conducted based on published data from Meng et al. (2021) which indicated an *n* = 6 was sufficient to observe the effects of RRS on mechanical hypersensitivity post paw incision surgery.

#### The effect of RRS on aversive- and anxiety-like behaviour post-surgery

Rats were exposed to RRS (6 h/day 21day) or no stress, followed by paw incision or sham surgery. Animals were tested in a battery of behavioural aversive or affective tests in the following order (days post-surgery): place-escape avoidance paradigm (PEAP; day 2), open field test (OFT: day 4), elevated plus maze (EPM: day 6) and light-dark Box (LDB: day 10). Mechanical hypersensitivity was assessed throughout the post-surgical period (days 0–13).

#### Transcriptomic and molecular alterations in the dorsal horn of the spinal cord

Rats underwent RRS or no stress followed by paw incision or sham surgery. Von Frey and Hargreaves testing was conducted on Day 5 post-surgery, immediately after which animals were euthanised by live decapitation or terminal anaesthesia using sodium pentobarbital followed by perfusion and L4-L6 dorsal horn was carefully removed and stored for subsequent transcriptomic or immunohistochemical analysis.

#### The role of the spinal NLRP3/IL-1β pathway in RRS-induced exacerbation of post-surgical mechanical hypersensitivity and aversion

Rats underwent RRS or no stress followed by paw incision surgery. Animals received an intrathecal injection of either the NLRP3 antagonist MCC950 (20 μg/30 μl; R&D Systems, USA), IL-1 receptor antagonist (IL-1Ra) (30 μg/30 μl Anakinra; human recombinant IL-1Ra, Swedish Orphan Biovitrum) or corresponding vehicle (5% DMSO or Saline). The doses were selected based on the literature [39, 40] and on pilot studies demonstrating that these doses are not analgesic in animals subjected to paw incision in the absence of stress. Animals received intrathecal injections of MCC950, IL-1Ra, or the corresponding vehicle and were placed in the PEAP arena (day 2 post-surgery) or the von Frey apparatus (day 5 post-surgery) 15 min post-injection. Pain-related aversion was assessed for 30 min and mechanical hypersensitivity for 60 min post administration.

#### Determining if the effects of RRS on post-surgical sensory and affective pain responding are due to stress-induced glucocorticoid or β-adrenergic receptor activation

Animals received systemic administration of the glucocorticoid receptor antagonist RU486 (10 mg/kg i.p. Merck, Ireland), propranolol hydrochloride (10 mg/kg i.p; Merck, Ireland) or vehicle (saline) 30 min prior to RRS or no stress for 21 days. The dosage and timing were based on previous studies [41, 42]. Despair-like behaviour (FST) was assessed 24 h following the last treatment (the morning following the last RRS exposure), after which all animals underwent paw incision surgery. Mechanical hypersensitivity was assessed on days 1–10, post-surgical aversion (PEAP) assessed on day 2 and anxiety-like behaviour (OFT) on day 4.

### Repeated restraint stress

In order to establish a model of stress-induced persistent post-surgical pain, we employed three different protocols of Repeated Restraint Stress (RRS). Rats were placed in cylindrical plastic restraining tubes for 3 days (6 h/day), 14 days (2.5 h/day) or 21 days (6 h/day). Each RRS session commenced 1–2 h after the onset of the light phase. The choice of restraint period was based on pilot studies and the literature demonstrating that these protocols elicit stress-induced alterations in behaviour and/or modulate post-surgical hypersensitivity [22, 43].

### Plantar hind paw incision

The plantar hind paw incision surgery was conducted in accordance with previous published papers [7]. Briefly, animals were anaesthetised with isoflurane. The plantar surface of the left hind paw received a 1-cm longitudinal incision through skin and fascia and the plantaris muscle was lifted and incised longitudinally. After haemostasis, the skin was closed with two 5–0 silk mattress sutures (ETHICON, San Lorenzo, Puerto Rico, USA) and rats were placed in a heated clean cage until recovery. The animals subjected to sham surgery underwent isoflurane anaesthesia for an equivalent time (10–15 min).

### Behavioural testing

#### Von Frey test

In order to assess mechanical sensitivity, paw withdrawal thresholds (PWT, g) were determined using an electronic von Frey (IITC, CA, U.S.A.). After a 20-min habituation to the testing apparatus, the stimulus was applied to the mid outer part of the incisional site of the rats. Two PWTs were taken per paw, which were then averaged for statistical analysis.

#### Hargreaves’ test

Immediately following Von Frey testing, heat sensitivity (paw withdrawal latency, PWL, s) was assessed using the Hargreaves’ test (IITC, CA, USA). After a 10-min habituation to the testing apparatus, PWLs were measured three times/paw with a cutoff of 20 s and the average of the three recordings was used for statistical analysis.

#### Forced swim test

The two-day FST took place 2 h after the last RRS session, and the following day as previously described [44]. The behavioural response of the rats (immobility) was assessed blindly by a trained observer with the aid of Ethovision 15.0 XT (Noldus Technology, Wageningen, The Netherlands).

#### Open field test

The open field test (OFT) was conducted as previously described [45]. The open field apparatus consisted of a circular arena (diameter 75 cm) with high walls (40 cm in height) and an inner bright central arena (diameter 40 cm). Each rat was placed in the centre of the arena and allowed to freely explore for a duration of 5 min. The time spent in the central zone was recorded using Ethovision 15.0 XT software package (Noldus Technology, Wageningen, The Netherlands).

#### Elevated plus maze

The elevated plus maze (EPM) test was conducted as previously described [45]. Each rat was placed in the central platform facing one of the open arms (75 lux) and allowed to explore the maze for 5 min. The time spent in the open arms was recorded and analysed using the Ethovision 15.0 XT software package (Noldus Technology, Wageningen, The Netherlands).

#### Light-dark box

Light/dark box (LDB) test was conducted as previously described [46]. The apparatus consisted of two connected compartments (40 cm × 40 cm x 60 cm) either brightly lit (1000 lux) or dark ( <10 lux). On the test day, rats were placed in the dark compartment at the start of the trial, and their behaviour was recorded in the test arena for 5 min using a video camera positioned above the apparatus. Ethovision 15.0 XT software package (Noldus Technology, Wageningen, The Netherlands) was used to analyse the time spent in each compartment.

#### Emotionality z-scores

To evaluate emotional dysregulation across multiple tests, a composite emotionality Z-score was calculated using data from the OFT, EPM and LDB. PEAP was not included in the Z-score calculation, as it reflects a measure of affective response to a painful stimulus rather than a direct measure of emotional dysregulation related to pain. For each test, raw scores (e.g., time spent in the centre of the arena, time spent in the open arms, time spent in the bright compartment) were converted into Z-scores to standardize the data across measures. The Z-scores were computed as follows: Z = (X−μ) /σ, where X is the individual score from a given test, μ is the mean of the scores within the control group and σ is the standard deviation of the control group [47]. Z-scores from each behavioural test were then averaged to generate an emotionality score for each animal, reflecting the overall level of emotional behaviour.

#### Place escape/avoidance paradigm

The affective component of pain or pain-related aversion was assessed using the Place Escape/Avoidance Paradigm (PEAP) [48]. In brief, the PEAP apparatus consisted of a brightly lit (1000 lux) and dark chamber (40 cm x 40 cm x 60 cm each compartment). Rats were placed in the dark compartment and allowed to freely explore both compartments for a total duration of 30 min during which time a 15 g Von Frey filament was applied to either the ipsilateral (when in the dark compartment) or contralateral (when in the light compartment) paw every 15 s. Time spent (s) in the bright compartment, was recorded and used as an indicator of the aversive response to mechanical stimulation.

### Corticosterone level measurement

Faecal samples were collected from individual rats 2–3 h following the last RRS session reflecting the peak faecal corticosterone level post- stress [49]. Corticosterone levels were quantified using the Enzo Life Sciences Corticosterone ELISA Kit (Enzo Life Sciences, Farmingdale, NY) following the manufacturer’s instructions. Results were expressed as ng/g.

### Bulk 3’RNA-seq and analysis

RNA was isolated from ipsilateral dorsal horn of the spinal cord using the Nucleospin® RNA II total isolation kit (Macherey-Nagel, Germany). RNA was extracted separately from 4 random individual animals per group, and each sample was processed and sequenced independently. Integrity Number (RIN) values of the samples used are presented in Supplementary Methods Table S1. The 3’RNA-Seq process used is an in-house adaptation of the method developed by Foley and colleagues [50], performed by IntegraGen, Paris, France. Full description is provided in the Supplementary Methods.

### RT-qPCR

RNA isolated from the ipsilateral and contralateral dorsal horn of the spinal cord was reverse transcribed into complementary DNA (cDNA) using a high-capacity cDNA synthesis kit (Applied Biosystems, Warrington, UK). For quantitative RT-PCR (RT-qPCR), each reaction well contained 1 μl of cDNA, 5 μl of SYBR Green (Applied biosystems, Warrington, UK), 3.5 μl of RNase-free H_2_O, and 0.5 μl of gene-specific primers. Results were normalized to *B-actin* and *Gapdh*, expressed as a percentage of the control group (no RRS + sham) and calculated using the ΔΔCT method. Primer sequences are listed in Supplementary Methods Table S2.

### Immunohistochemistry for Iba1

Rats were transcardially perfused with phosphate buffer (PB) and 4% paraformaldehyde (PFA) in PB, and the spinal cords were post-fixed overnight at 4 °C followed by storage in 20% sucrose. The lumbar spinal cord segments (L4–L6) were sectioned at 30 µm, blocked in 5% goat serum, incubated overnight with rabbit anti-Iba1 (1:1000, WAKO, 019-19741), washed and incubated with secondary antibody conjugated to Alexa Fluor 594 (1:500).

Fluorescent images were acquired using a Zeiss Axio Imager 2 microscope. Maximum intensity projections of z-stacks and tile scans were generated using Zen Blue software (ZEISS Microscopy Software) to visualize and analyse high-resolution image datasets. Image acquisition settings, including objective magnification (40x), exposure time (100 ms), and z-stack step size (2 µm), were kept consistent across samples. For subsequent analyses, 3–4 sections per animal were used. Iba1-positive cells were quantified using Imaris 10.1 (Oxford Instruments). Fluorescence intensity-based analyses were performed in ImageJ (Fiji) by measuring mean pixel intensity in manually defined regions of interest (ROIs) within the ipsilateral and contralateral dorsal horn. ROIs were determined based on anatomical landmarks of the rat spinal cord, guided by the spinal cord atlas. Specifically, we focused on the superficial dorsal horn (laminae I–III), which was outlined consistently across sections. Background subtraction and normalization methods were applied to ensure accurate quantification.

### Statistical analysis

Data was analysed using GraphPad Prism, version 9.0 (GraphPad Software, Inc., La Jolla, CA, USA), which was also used to generate graphs. Prior to statistical testing, all datasets were assessed for normality and homogeneity. Comparisons between two groups, such as for FST and faecal corticosterone data, were performed using unpaired student’s two-tailed t-test. Data from von Frey and Hargreaves’ tests were examined using two-way repeated measures ANOVA followed by Newman-Keuls post-hoc test where appropriate. Behavioural tests including the PEAP, OFT, EPM, LDB and emotionality z-scores were evaluated with two-way ANOVA followed by Newman-Keuls post-hoc test where appropriate. Changes in gene expression (qPCR), microglia numbers and fluorescence intensity in the dorsal horn were analysed using three-way ANOVA followed by Newman-Keuls post hoc test. For clarity, the results of the ANOVA comparisons are presented in Supplementary Table S3. Statistical significance was set at *p* <0.05, and results are expressed as group means ± standard error of the mean (SEM).

## RESULTS

### RRS elicits a depressive-like phenotype and increases the magnitude and duration of mechanical and heat hypersensitivity post-surgery

**Fig. 1:**
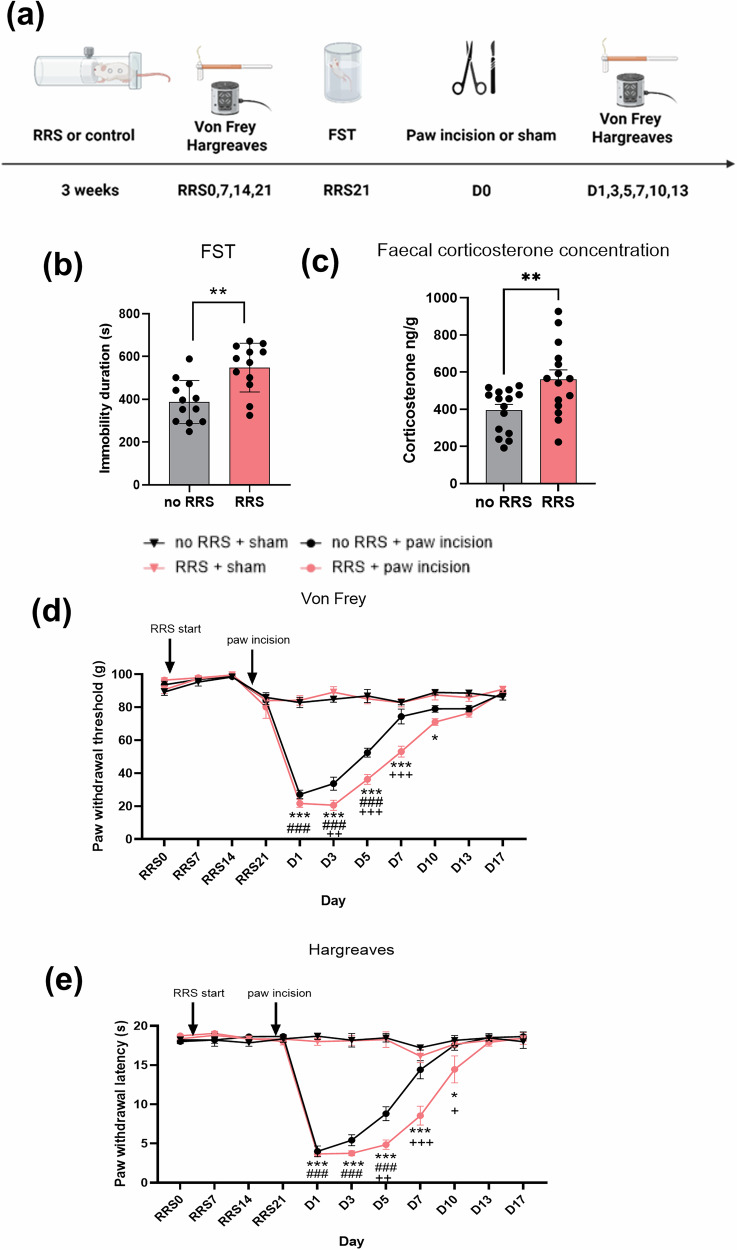
RRS elicits a depressive-like phenotype and increases the magnitude and duration of mechanical and heat hypersensitivity post-surgery. **a** Experimental design. The effect of RRS on (**b**) immobility time in the FST (**p <0.01) **c** faecal corticosterone levels (**p <0.01), **d** mechanical (von Frey) and **e** thermal heat sensitivity (Hargreaves test) during RRS and post-paw incision or sham surgery. ^###^*p* <0.001 no RRS + sham vs. no RRS + paw incision, *p <0.05, ***p <0.001 RRS + sham vs. RRS + paw incision, ^+^*p* <0.05, ^++^*p* <0.01 ^+++^*p* <0.001 no RRS + paw incision vs RRS + paw incision. *n* = 6/group.

While all RRS protocols elicit a decrease in body weight gain (Supplementary Fig. S2) only RRS for 6 h/day for 21 days resulted in a depressive-like phenotype and increased the magnitude and duration of post-surgical nociceptive responding (Fig 1). Data pertaining to the other RRS protocols employed are presented in Supplementary Figs. S3 and S4. RRS (6 h/day for 21 days) significantly increased immobility time (*t*(22) = 3.666, *p* <0.01) in the FST (Fig. 1b) and faecal corticosterone levels were significantly elevated compared to non-RRS counterparts (*t*(28) = 2.847, *p* <0.01) (Fig. 1c), confirming an altered stress response and depressive-like phenotype of animals subjected to this RRS protocol.

Analysis revealed that RRS did not alter paw withdrawal thresholds (PWTs) (Fig. 1d) or paw withdrawal latencies (PWLs) (Fig. 1d) at any point throughout the RRS period (days 0, 7, 14, 21). PWTs and PWLs were significantly reduced in rats that received paw incision compared to sham groups, indicating the development of post-surgical mechanical and heat hypersensitivity (Fig. 1d, e). RRS exacerbated post-surgical mechanical hypersensitivity on days 3, 5 and 7 and prolonged mechanical hypersensitivity to day 10 post-surgery compared to non-RRS counterparts, which returned to baseline by day 7 (Fig. 1d). RRS also exacerbated post-surgical heat hypersensitivity on days 5 and 7 post-surgery (Fig. 1e) and prolonged heat hypersensitivity to day 10 compared to non-RRS counterparts. Taken together, these results demonstrate that RRS exacerbates and prolongs mechanical and heat hypersensitivity post-surgery.

### RRS increases pain-related aversion and anxiety-like responding post-surgery

**Fig. 2:**
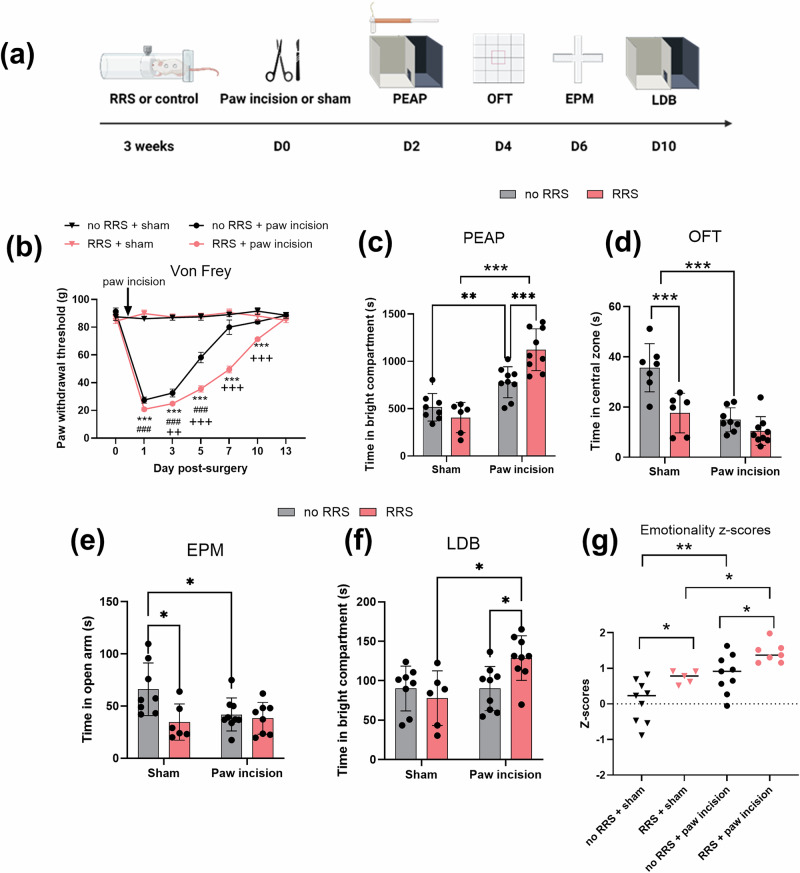
RRS increases pain-related aversion and anxiety-like responding post-surgery. **a** Experimental design. The effect of RRS on (**b**) paw withdrawal threshold (von Frey) ^###^*p* <0.001 no RRS + sham vs. no RRS + paw incision, ***p <0.001 RRS + sham vs. RRS + paw incision, ^++^*p* <0.01 ^+++^*p* <0.001 no RRS + paw incision vs RRS + paw incision. **c** Pain-related aversion (PEAP) and anxiety like behaviour in the (**d**) open field test (OFT), **e** elevated plus maze (EPM) and **f** light dark box (LDB). **g** Overall emotionality (z-score). *n* = 6–8 per group (*p <0.05, ***p <0.001, ***p <0. 001).

A series of behavioural tests was conducted to evaluate the emotional component of post-surgical pain and whether RRS alters this state. The behavioural assays used (PEAP, OFT, EPM and LDB) primarily assess exploratory and aversive behaviours, which are commonly interpreted as proxies for affective-like responses in rodents. Locomotor activity was assessed in these tests (Supplementary Fig. S5) and was not found to differ between groups, indicating that the observed behavioural changes are not related to alterations in general mobility, but rather reflect specific differences in affective or pain-related responses. On Day 2 post-surgery, animals that underwent surgery spent increased time in the aversive bright compartment of the PEAP test, an effect further increased by prior RRS (Fig. 2c). In the OFT (day 4 post-surgery), animals subjected to RRS (no surgery) spent less time in the central zone compared to non-stressed counterparts (Fig. 2d). Similarly, animals that underwent surgery, whether exposed to RRS or not, also spent less time in the central zone of the OFT. In the EPM (day 6 post-surgery), RRS or paw incision alone decreased time spent in the open arms, but their combination did not further modify this response (Fig. 2e). On day 10 post-surgery, animals exposed to RRS + paw incision spent more time in the bright compartment of the LDB, compared to rats that underwent RRS or surgery alone (Fig. 2f). Z-scores were calculated to examine overall effect on emotionality and revealed that RRS and paw incision alone increase emotionality, an effect further heightened in animals subjected to RRS + surgery (Fig. 2g). Together, these findings demonstrate that RRS exacerbates both the sensory and the affective components of post-surgical pain and amplifies pain-related aversion.

### RRS induces transcriptional alterations in the spinal NLRP3-IL-1β pathway post-surgery

**Fig. 3:**
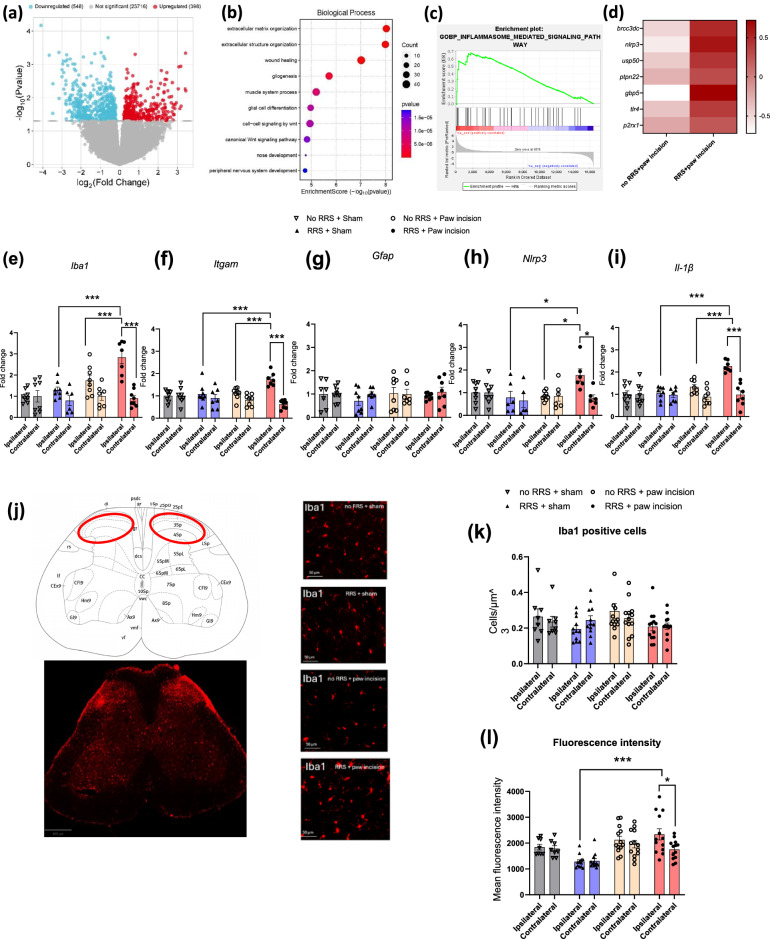
RRS induces transcriptional alterations in the spinal NLRP3-IL-1β pathway post-surgery. **a** Volcano plot depicting the 946 genes differentially expressed (nominal *P* values <0.05) between no RRS + paw incision (*n*  =  4) and RRS + paw incision (*n*  =  4) groups. **b** Top biological processes in the Gene Ontology pathway enrichment analyses of differentially expressed genes. **c** Gene set enrichment analysis (GSEA) significantly enriched gene set, *p* value = 0.005. **d** Upregulation of NLRP3 inflammasome pathway-related genes *(Brcc3dc*, *P*  =  0.094;*Nlrp3*, *P*  =  0.071; *Usp50*, *P*  =  0.163; *Ptpn22*, *P*  =  0.507; *Gbp5*, *P*  =  0.074; *Tlr4*, *P*  =  0.43; *P2rx1*, *P*  =  0.027. Z-scores were calculated for each gene across all samples to standardize relative expression levels. Relative gene expression of (**e**) *Iba1*, **f** *Itgam*, **g** *Gfap*, **h** *Nlrp3* and **i** *Il-1β* in the ipsilateral dorsal horn, (*n* = 5–8 per group). **j** Representative photomicrographs of Iba1 positive staining (red) in the spinal cord and in the ipsilateral dorsal horn for each of the 4 groups: no RRS + sham (top left), RRS + sham (top right), no RRS + paw incision (bottom left) and RRS + paw incision (bottom right). **k** The number of Iba1-positive cells and (**l**) mean fluorescence intensity of Iba1 staining in the region of interest (red circle) from ipsilateral and contralateral dorsal horn. *p <0.05, ***p <0.001.

RNAseq was used to examine the molecular mechanisms underlying RRS-induced alterations in post-surgical pain. At a nominal *p*value threshold (*p* <0.05), 946 genes were found to be significantly differentially expressed in the RRS + paw incision group compared to no RRS + paw incision controls (Fig. 3a). Gene Ontology (GO) analysis highlighted significant enrichment of terms related to wound healing (*Tnf, P2rx1, Wnt4, C1qtnf1* etc), gliogenesis and glial cell differentiation *(Nfib, Bmerrb1, Hexb, Nrros, Ctnnb1* etc) (Fig. 3b), suggesting a potential role of neuroimmune mechanisms in the observed phenotype. Gene Set Enrichment Analysis (GSEA), a significant positive enrichment of the inflammasome-mediated signalling pathway (normalized enrichment score = 1.69, *p* <0.05, FDR <0.1) in RRS + paw incision rats (Fig. 3c). Notable upregulated genes in the RRS + paw incision vs no RRS + paw incision group included *Nlrp3*, *Tlr4*, *P2rx1*, and other genes mediating activation of inflammasome signalling (Fig. 3d). Further analysis on the effects of RRS, paw incision and their combination on the transcriptomic profile of the spinal cord are presented in Figs. S6, S7, S8 and S9.

RT-qPCR analysis revealed that RRS + paw incision was associated with significant upregulation of the expression of microglial markers *Iba1* and *Itgam* (Fig. 3e, f), and *Nlrp3* and *Il-1β* (Fig. 3g, h), but not *Gfap* (Fig. 3i), compared to non-stressed counterparts. Immunohistochemical analysis of Iba1 staining confirmed an overall effect of RRS (*F*_(1,84)_ = 5.401, *p* = 0.0225) on the number of Iba1-positive cells, although *post hoc* analysis failed to reveal a significant difference between the groups (Fig. 3k). Analysis of fluorescence intensity revealed an increase in Iba1 levels in the ipsilateral dorsal horn of RRS + paw incision group compared to the contralateral side and animals exposed to RRS alone (Fig. 3l). Together, these findings indicate the exacerbation of post-surgical pain in RRS rats is accompanied by increased Iba1 immunoreactivity and the upregulation of NLRP3-IL-1β inflammasome pathways.

### Blockade of spinal NLRP3 or IL-1β signalling attenuates RRS-induced exacerbation of pain-related aversion and mechanical hypersensitivity post-surgery

**Fig. 4:**
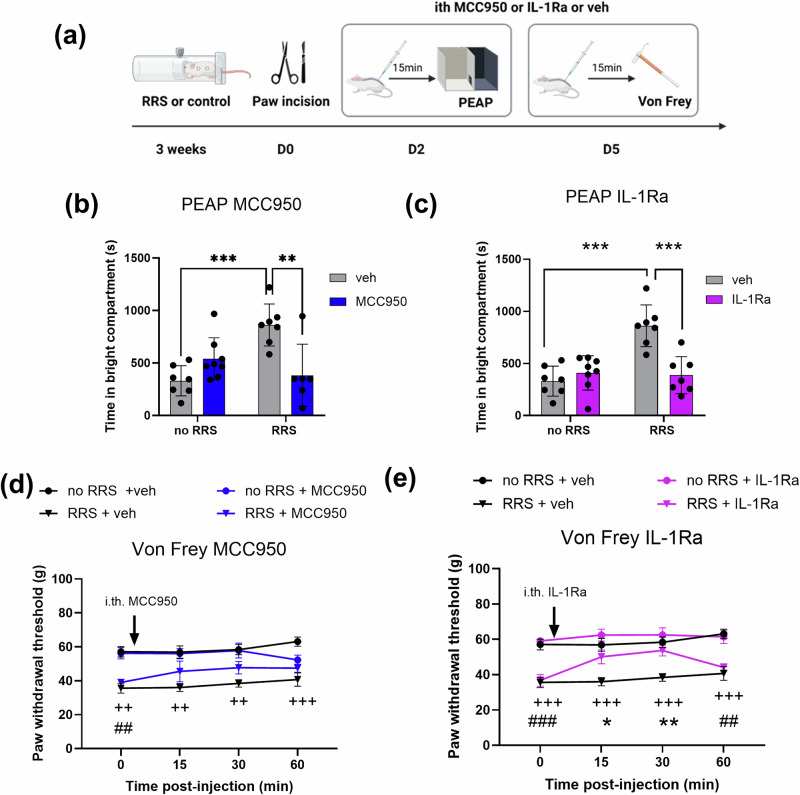
Blockade of spinal NLRP3 or IL-1β signalling attenuates RRS-induced exacerbation of pain-related aversion and mechanical hypersensitivity post-surgery. **a** Experimental design. The effect of intrathecal administration of MCC950 and IL-1Ra on (**b**, **c**) pain-related aversion (PEAP; day 2 (**P <0.01 ***P <0.001) and **d**, **e** paw withdrawal thresholds (von frey; Day 5) following paw incision surgery. ^++^*p* <0.01, ^+++^*p* <0.001 No RRS + veh vs RRS + veh, ^##^*p* <0.01, ^###^*p* <0.001 no RRS + IL-1Ra/MCC950 vs RRS + IL-1Ra/MCC950, *p <0.05, **p <0.01 RRS + veh vs RRS + IL-1Ra. *n* = 6–8 per group.

Using a pharmacological approach, we examined the role of spinal NLRP3-IL-1β pathway in the behavioural effects observed. As previously observed, pre-exposure to RRS increased the time spent in the bright compartment of the PEAP on day 2 post-surgery (Fig. 4b, c). Intrathecal administration of the NLRP3 inhibitor MCC950 (Fig. 4b) or IL-1Ra (Fig. 4c) attenuated the RRS-induced increase in time spent in the bright chamber of the PEAP chamber.

On day 5 post-surgery, our data re-confirmed that RRS exacerbates mechanical hypersensitivity (Fig. 4d, e). Intrathecal administration of MCC950 (Fig. 4d) or IL-1Ra (Fig. 4e) did not alter paw withdrawal thresholds of non-stressed paw incision animals. However, IL-1Ra significantly attenuated surgery-induced mechanical hypersensitivity in animals pre-exposed to RRS compared to vehicle counterparts. No significant difference was observed between no RRS and RRS groups that received MCC950. These data suggest that increased spinal NLRP3-IL-1β signalling mediates RRS-induced exacerbation of pain-related aversion and mechanical hypersensitivity post-surgery.

### Chronic propranolol, but not RU486, attenuates RRS-induced exacerbation of post-surgical hypersensitivity, pain-related aversion and anxiety behaviour

**Fig. 5:**
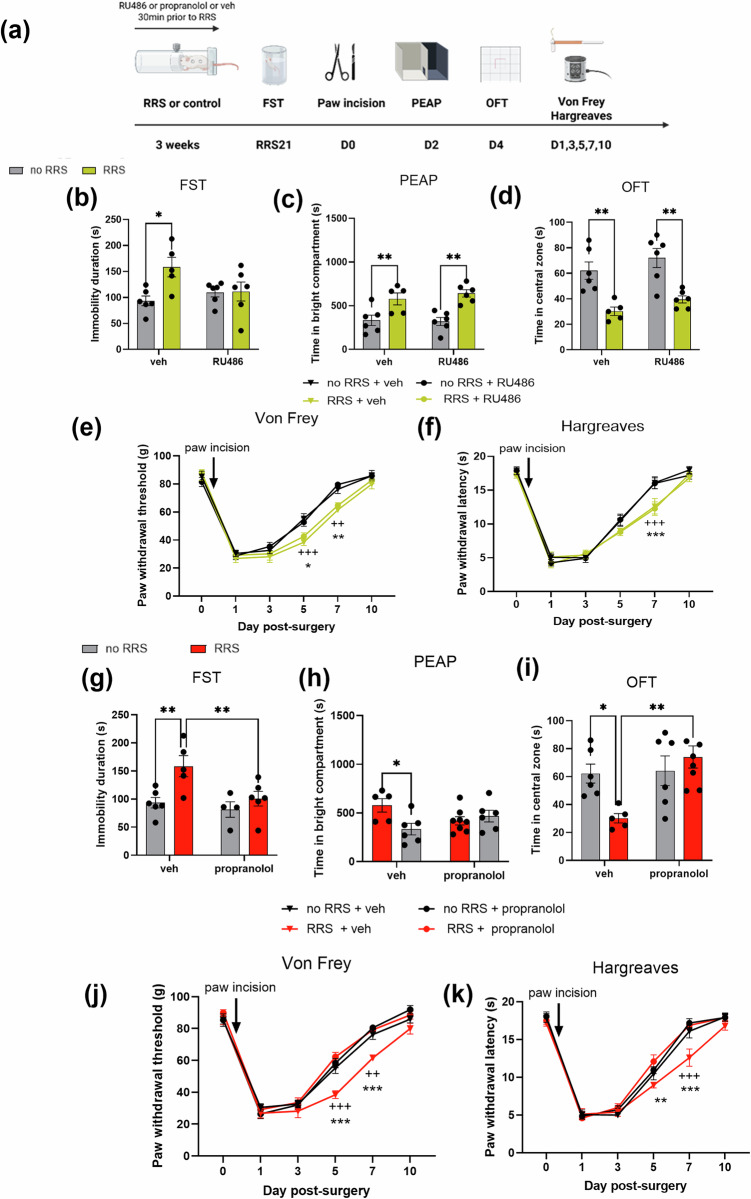
Chronic propranolol, but not RU486, attenuates RRS-induced exacerbation of post-surgical hypersensitivity, pain-related aversion and anxiety behaviour. **a** Experimental design. The effect of chronic administration of glucocorticoid antagonist RU486 (21 days) on (**b**) immobility in the FST (*p <0.05), **c** pain-related aversion (PEAP Day 2 post surgery; **P <0.01) **d** time in the centre zone of the OFT (Day 4 post surgery, **P <0.01), **e** paw withdrawal threshold (von Frey) and **f** paw withdrawal latency (Hargreaves test). ^++^*p* <0.01, ^+++^*p* <0.001 no RRS + paw incision + veh vs RRS + paw incision + veh, *p <0.05, **p <0.01, ***p <0.001 no RRS + paw incision + RU486 vs RRS + paw incision + RU486. The effect of chronic administration of propranolol (21 days) on (**g**) immobility in the FST (**p <0.01), **h** pain-related aversion (PEAP Day 2 post surgery; *P <0.05) **i** time in the centre zone of the OFT (Day 4 post surgery, *P <0.05 **P <0.01), **j** paw withdrawal threshold (von Frey) and **k** paw withdrawal latency (Hargreaves test). ^++^*p* <0.01, ^+++^*p* <0.001 no RRS + paw incision + veh vs RRS + paw incision + veh, *p <0.05, **p <0.01, ***p <0.001 RRS + paw incision + veh vs RRS + paw incision + propranolol. *n* = 5–8 per group.

Chronic administration of the glucocorticoid receptor antagonist RU486 prior to each RRS exposure prevented the RRS-induced increase in immobility in the FST (Fig. 5b), suggesting that blockade of glucocorticoid signalling can prevent RRS-induced increase in depressive-like behaviour. Post-surgery, there was no effect of RU486 on time spent in the bright compartment of the PEAP (Fig. 5c), time spent in the central zone of the OFT (Fig. 5d), PWTs (Fig. 5e) and PWLs (Fig. 5f) in either RRS or no RRS exposed animals.

Propranolol prevented the RRS-induced increase in immobility in the FST (Fig. 5g), suggesting that RRS-induced β-adrenergic activation contributes to the depressive-like phenotype observed. Post-surgery, propranolol blocked the RRS-induced increase in the time spent in the bright compartment of the PEAP arena (Fig. 5h) and the decrease in the time spent in the centre of the OFT arena (Fig. 5i). In addition, RRS-exposed rats that received propranolol exhibited increased paw withdrawal thresholds (Fig. 5j) and paw withdrawal latencies (Fig. 5k) compared to their vehicle-treated counterparts on post-surgical days 5 and 7. These data suggest that RRS-induced exacerbation of sensory and affective post-surgical pain is likely mediated by enhanced β-adrenergic, but not glucocorticoid, receptor activity.

## Discussion

Psychological factors such as chronic stress and depression are associated with increased and prolonged post-surgical pain, increased analgesic requirement and well recognised risk factors for the development of chronic post-surgical pain. This study demonstrated that chronic stress in the form of RRS results in altered stress responsivity and a depressive-like phenotype and exacerbates and prolongs sensory and affective pain responding post-surgery. Our data suggest that these behavioural effects are mediated by stress-induced β-adrenergic activity and enhanced spinal microglial Iba1 immunoreactivity and NLRP3-IL-1β signalling. These data provide further insight into the molecular and cellular mechanisms mediating the effects of chronic stress and negative affect on post-surgical pain.

We showed that repeated restraint stress (RRS) for 6 h/day for 21 days induces both behavioural and physiological changes consistent with a depressive-like state. The term *“*depressive-like behaviour” is used here, as in the wider literature [34, 51], to describe measurable behavioural outcomes in rodents (such as increased immobility in the FST or reduced sucrose preference) that reflect specific facets of human depressive symptomatology rather than the full clinical syndrome. Specifically, we observed reduced body weight gain, increased immobility in the FST and elevated faecal corticosterone levels, mirroring prior studies that demonstrated similar effects following 21 days of RRS (6 h/day) [22, 52, 53]. These results collectively underscore that prolonged RRS exposure not only elicits a depressive-like phenotype but also disrupts physiological homoeostasis, highlighting a broader dysregulation of the stress response system.

Previous studies have reported that RRS alters mechanical withdrawal thresholds in the absence of injury. For example, RRS for 3 (6 h/day) [22, 43] or 4 weeks (2 h/day) [54, 55] in rats and 2 [56], 3 [57] or 4 weeks [58] in mice (6 h/day) resulted in significant increases in mechanical and heat sensitivity, indicative of stress-induced hypersensitivity. In comparison, in the present study there was no effect of RRS on pre-surgical mechanical or heat sensory thresholds over the course of the 21 days of RRS. This discrepancy may stem from differences in the time of testing relative to stress exposure. For example, Imbe et al. measured mechanical sensitivity in rats 1 h after RRS exposure, while we assessed PWTs 18–20 h post-RRS to capture the broader, cumulative effects of RRS sessions rather than any immediate/acute effects of RRS. Alternatively, or in addition, differences in the rat strain and supplier of animals could contribute to different outcomes.

Regarding the impact of RRS on post-surgical somatosensory responses, our findings indicate that RRS significantly heightened mechanical and heat hypersensitivity, particularly on post-surgical days 5 and 7, and prolonged hypersensitivity up to day 10. These results align with the published literature demonstrating that both acute and chronic pre-surgical stressors amplify the magnitude and prolong the duration of post-surgical somatosensory hypersensitivity in rodents [23]. In particular our data align with that reported by, Meng and colleagues who demonstrated 21-day RRS protocol (6 h/day) prolongs mechanical hypersensitivity up to day 10 following paw incision [22]. However, to our knowledge, this is the first study to demonstrate that RRS also results in post-surgical heat hypersensitivity and enhanced affective pain responding. Notably, we found that paw-incised rats exhibited significant aversion to mechanical stimuli (PEAP) as early as two days post-surgery. Comparable findings have been reported in a rat model of inguinal hernia repair, where animals demonstrated increased pain-related aversion within just three hours post-surgery [59]. Interestingly, post-surgical pain-related aversion was further increased in rats subjected to prior RRS. These results suggest that RRS may increase unpleasantness and emotional distress, leading to an exaggerated avoidance of noxious stimuli compared to non-stressed paw-incised counterparts.

Regarding anxiety-like behaviour post-surgery, we observed that rats that underwent paw incision (without RRS) displayed anxiety-like behaviour on day 4 (OFT test) and day 6 post-surgery (EPM test) as well as an overall increase in emotionality score, data which align with previous studies showing that paw incision can induce anxiety-like behaviour between 1 h to 5 days post-surgery [8, 9]. Interestingly, prior RRS did not further enhance the anxiety-related behavioural phenotype observed post-surgery, despite an exacerbation and prolongation of the somatosensory hypersensitivity. It is possible that this may be due to limitations of the behavioural tests, such as a potential floor effect, such that further increases in anxiety-related behaviour could not be detected. However, assessment of emotionality score across tests revealed a surgery-induced increase, which was further exacerbated by prior RRS. Thus, taken together, prior chronic stress or a negative affective state is associated with enhanced and prolonged sensory and affective pain responding post-surgery.

It is well-documented that surgical procedures such as paw incision activate neuroinflammatory pathways, contributing to pain hypersensitivity [20, 24, 60–68]. Previous studies have shown that alterations in spinal microglia, including changes in morphology and cytoplasmic and cell-surface marker expression, contribute to paw incision-induced hypersensitivity for up to 4 days post-surgery, coinciding with active hypersensitivity [24, 63, 66, 67]. Interestingly, in the current study, paw incision in the absence of prior RRS was not associated with any change in spinal microglial activity/density or the expression of proinflammatory markers 5 days post-surgery. Data suggest that as sensory hypersensitivity post-injury resolves, microglia no longer exhibit detectable morphological or marker changes. [20, 63, 66, 67] and other glial and neuroimmune factors may come into play. Accordingly, on day 5 post-surgery, while animals still display mechanical and heat hypersensitivity, the magnitude of this response is reduced compared to earlier timepoints post-surgery. This is not the case for animals pre-exposed to RRS which continue to display robust sensory hypersensitivity and associated changes in microglial reactivity and proinflammatory mediator expression. This heightened neuroimmune and glial activity in animals pre-exposed to stress was further confirmed by transcriptomic analysis revealing alterations in inflammatory pathways, gliogenesis and glial cell differentiation.

Significant enrichment of the spinal NLRP3-IL-1β pathway in the ipsilateral dorsal horn of rats subjected to both RRS and paw incision was confirmed by RT-qPCR, highlighting this as a potential key mediator of the effects of RRS on post-surgical pain. Similarly, increased Iba1 immunoreactivity [20] accompanied by increased IL-1β [11, 24] have been associated with the effects of acute stress on post-surgical mechanical hypersensitivity and perioperative intrathecal inhibition of microglia decreased *Il-1β* expression and shortened the duration of SDS-induced post-surgical mechanical hypersensitivity [11]. In the present study, modest changes ( ~ 2-fold) in Nlrp3 and Il-1b expression and IBA1 intensity in the dorsal horn were observed. It is important to note that these findings could reflect subtle, physiological alterations consistent with parainflammation, in which microglia and other immune components adopt an intermediate, adaptive state in response to stress or injury [25, 26], rather than a full canonical neuroinflammatory response. Our RNAseq data further indicate that many differentially expressed genes are involved in cellular remodelling, including cytoskeletal dynamics, cell signalling, and synaptic regulation (Figure S6), supporting the suggestion that chronic stress and injury may induce microglial and neuronal adaptations alternative to classical inflammation.

Glucocorticoid receptor inhibition attenuates acute immobilization stress-induced prolongation of hypersensitivity to mechanical, heat, and cold stimuli in male rats [17]. Similarly, glucocorticoid receptor inhibition mitigated the SPS-induced exacerbation of post-surgical mechanical hypersensitivity, microglial alterations and cytokine expression [12]. In contrast, the data herein revealed that while glucocorticoid receptor inhibition prevented the RRS-induced increase in despair-like behaviour, it did not prevent the exacerbation of post-surgical somatosensory hypersensitivity, aversion, or anxiety-like behaviour. These data suggest that pre-surgical despair-like behaviour is not a prerequisite for RRS-induced exacerbation of post-surgical somatosensory hypersensitivity, aversion, or anxiety-like behaviour. Taken together the data suggest that acute stress-induced exacerbation of post-surgical pain my involve glucocorticoid receptor signalling, while the effects of chronic stress may be mediated through alternative mechanisms. In comparison, antagonism of β-adrenergic receptors prevented the RRS-induced increase in despair-like behaviour, as well as the exacerbation of hypersensitivity, pain-related aversion and anxiety-like behaviour post-surgery. Our findings are in line with recent research suggesting that serum IL-6, induced by β3-adrenoceptor signalling in brown adipocytes, can lead to blood-spinal cord barrier disruption and enhanced spinal Iba1 immunoreactivity, potentially contributing to acute stress-mediated persistent post-surgical hypersensitivity [69]. β-adrenergic receptors are known to induce microglial priming, and propranolol inhibits restraint stress-induced marker changes in microglia [70]. Given that IL-6 and IL-1β are both key neuroimmune mediators involved in stress-induced changes of microglial state and pain modulation, we propose that that chronic stress primes spinal microglia via β-adrenergic receptor signalling, and when combined with paw incision, this leads to upregulation of the NLRP3-IL-1β signalling, driving both sensory and affective pain exacerbation. Future studies will examine this in detail to identify the sites and mechanisms through which β-adrenergic receptors modulate the RRS-induced exacerbation of post-surgical pain responding.

We acknowledge that a limitation of the present study is the use of male rats only. Well-documented sex-specific neuroimmune mechanisms have been demonstrated to underlie the development of neuropathic pain [71, 72], however it is unknown if similar mechanisms underlie the stress-induced prolongation of post-surgical pain. Our pilot data suggest that although both sexes display comparable RRS-induced prolongation of post-surgical mechanical and thermal hypersensitivity, there was no associated change in the expression of *iba1, nlrp3* or *il1b* in the dorsal horn of female rats, as was observed in male rats in this study. These data suggest possible sex-specific neurobiological mechanism which drive the behavioural changes observed, however we cannot rule out that changes may occur at a protein level or post transcriptional processing such as protein cleavage, phosphorylation, trafficking and degradation, which can occur independently of transcript expression. Future studies will focus on examining whether chronic stress combined with surgery engage distinct sex-dependent molecular mechanisms resulting in the prolongation of pain-related sensory and affective responding.

In conclusion, our findings demonstrate that RRS significantly exacerbates both somatosensory hypersensitivity and affective-pain dysregulation post-surgery, effects mediated, at least in part, by the β-adrenergic receptor activation, alterations in spinal microglia and NLRP3-IL-1β signalling. Together, these results provide valuable insights into how chronic stress and a negative affective state alters post-surgical pain processing and emotionality, suggesting potential targets for therapeutic interventions for the co-morbidity of mood and pain disorders.

## Supplementary information

Supplementary Methods, Figures and Analysis

Supplementary Table S3

## Data Availability

Statistical analysis is provided in Supplementary Table S3. Raw data are available from the corresponding author upon request.
