# Supplementary material for: Repeated restraint stress-induced increase in post-surgical somatosensory hypersensitivity and affective responding is mediated by β-adrenergic receptor activation and spinal NLRP3-IL1β signalling in male rats
Source: Neuropsychopharmacology. 2026 Jan 9;51(6):1032–44. doi: 10.1038/s41386-025-02305-x (PMC13125613; doi:10.1038/s41386-025-02305-x)
Supplement: Supplementary file 1 — Supplementary Methods, Figures and Analysis [file 41386_2025_2305_MOESM1_ESM.docx]

SUPPLEMENTARY MATERIAL

**SUPPLEMETRY METHODS**

**Experimental Design**

**
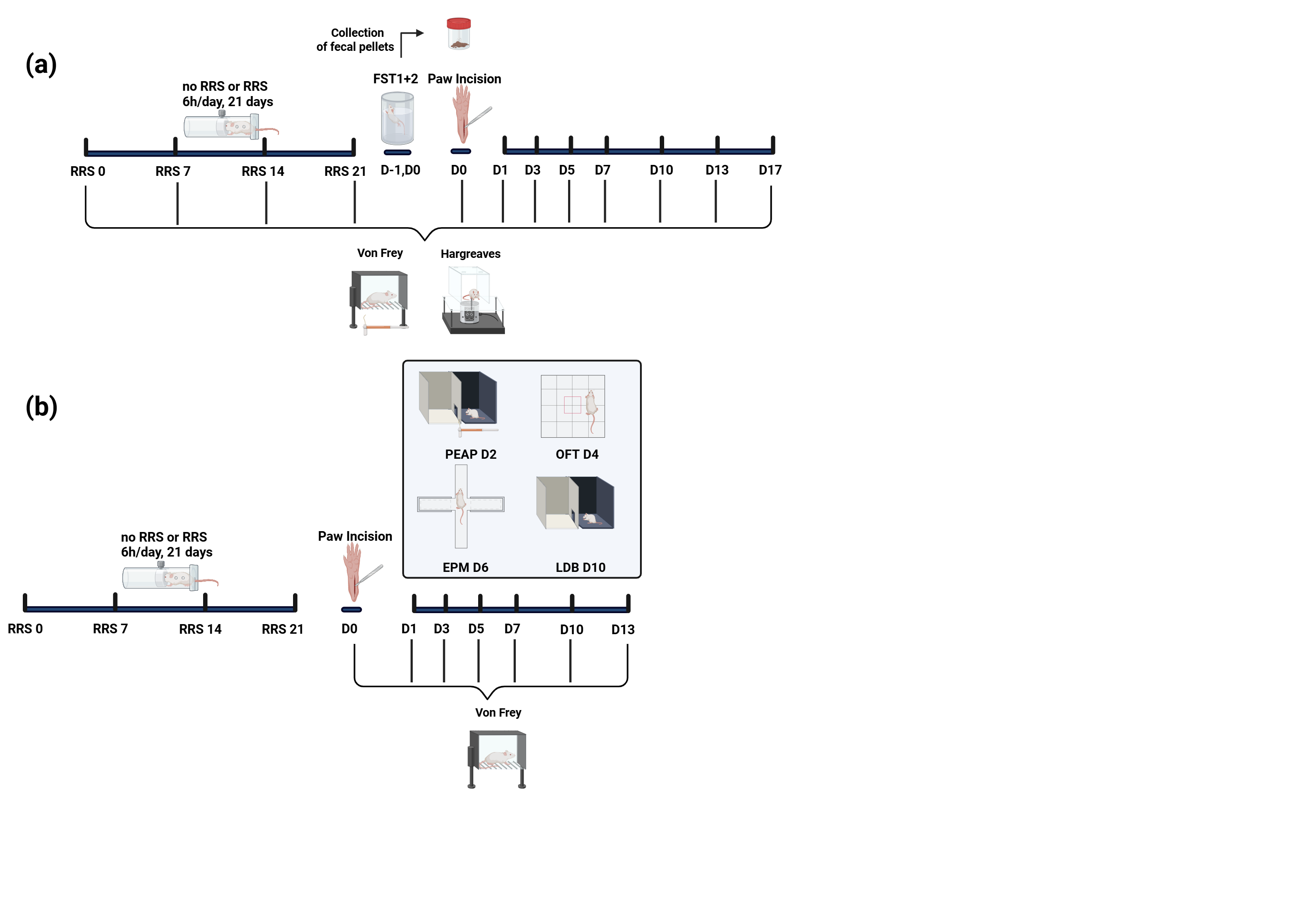
**

Figure S1: Experimental timeline for study 1 and 2 (created using Biorender.com)

**Bulk 3’RNA-seq and analysis**

RNA was isolated from ipsilateral dorsal horn of the spinal cord using the Nucleospin® RNA II total isolation kit (Macherey-Nagel, Germany). RNA was extracted separately from 4 individual animals per group, and each sample was processed and sequenced independently. RNA Integrity Number (RIN) values of the samples used are presented in Supplementary Table S1.

***Supplementary Table S1: RNA Integrity Number (RIN) of samples used for RNAseq.***

| **Sample name** | **RIN** | **Group** |
| --- | --- | --- |
| SC1 | 8.4 | No RRS + paw incision |
| SC2 | 6.9 | No RRS + paw incision |
| SC4 | 5.8 | RRS + sham |
| SC5 | 7.5 | RRS + paw incision |
| SC6 | 8.6 | RRS + paw incision |
| SC7 | 9.3 | No RRS + paw incision |
| SC8 | 7.5 | No RRS + paw incision |
| SC9 | 6.6 | RRS + sham |
| SC10 | 9.5 | RRS + sham |
| SC11 | 9 | No RRS + sham |
| SC12 | 7.3 | No RRS + sham |
| SC15 | 8.8 | RRS + sham |
| SC17 | 9.2 | RRS + paw incision |
| SC20 | 7.7 | No RRS + sham |
| SC21 | 9 | RRS + paw incision |
| SC24 | 9 | No RRS + sham |

The 3’RNA-Seq process used is an in-house adaptation of the method developed by Foley and colleagues (Foley et al., 2019), performed by IntegraGen, Paris, France. In summary, 10 ng of total RNA was fragmented and the 3’ ends of mRNA were captured using an RT primer containing a poly(T) sequence and a Unique Molecular Identifier (UMI). Illumina adapters were incorporated during this step via template switching. The resulting fragments underwent two rounds of PCR amplification to complete the Illumina adapters and add indexing sequences. The read structure is as follows: Read 1: Contains a 26-base UMI followed by the beginning of the poly(T) sequence. Read 2: Starts with a GGG sequence (to be trimmed), followed by the variable-length 3’ insert tag and the poly(A) tail. Sequencing was performed on an Illumina NovaSeq 6000 platform using a 2x100 bp paired-end configuration.

The bulk RNA-seq data analysis was performed using Galaxy (Galaxy, 2024). Preprocessing began using Trimmomatic, where adapter sequences were removed, and reads were trimmed if their quality scores fell below 20. Reads shorter than 35 base pairs were discarded. Quality control was subsequently performed using FastQC, ensuring all samples met the required standards. The reads were aligned to the rat reference genome (rn7) using HISAT2, and feature quantification was achieved with feature counts.

Downstream analyses, including normalization and differential expression analysis, were conducted using limma-voom. Differentially expressed genes (DEGs) were identified using the following criteria: a *p*-value threshold of 0.05 and absolute fold change cutoff of 1. Gene Ontology Enrichment Analysis was performed using SRplot (Tang et al., 2023). Finally, Gene Set Enrichment Analysis was performed using the GSEA software (Broad Institute) to identify enriched gene sets. Expression values of the selected genes were standardized to z‑scores and visualized in a heatmap. For each gene, the z‑score was calculated as follows: Z=(X−μ) /σ, where X is the expression value in an individual sample, μ is the mean expression across the control group, and σ is the standard deviation of expression values within that control group.

**RT-qPCR**

***Supplementary Table S2: Primer sequences***

| **Gene** | **Forward primer sequence** | **Reverse primer sequence** | **Origin** |
| --- | --- | --- | --- |
| *Iba1* | 5' -ATGTCCTTGAAGCGAATGCT-3' | 5' -TTCTCAAGATGGCAGATCTCTT-3' | (Luo et al., 2019) |
| *Itgam* | 5'-CAAGGAGTGTGTTTGCGTGT-3' | 5'-AGAAGGCTCGGACAACTGAG-3' | (Zhang et al., 2021) |
| *Il-1β* | 5'-TCCCGACCATTGCTGTTTCC-3' | 5'-TGAAGTCAACTATGTCCCG-3' | (Loram et al., 2010) |
| *Nlrp3* | 5'-AACTTGCAGAAGCTGGGGT-3' | 5'-GGTGCAGAAGTCCCTCACAG-3' | Designed in-house |
| *Gfap* | 5' -ACATCGAGATCGCCACCTAC-3' | 5' -ACATCACATCCTTGTGCTCC-3' | (Tomassoni et al., 2004) |
| *B-actin* | 5'-GTCGTACCACTGGCATTGTG-3' | 5'CTCTCAGCTGTGGTGGTGAA-3' | (Luo et al., 2019) |
| *Gapdh* | 5'AGACAGCCGCATCTTCTTGT-3' | 5'CTTGCCGTGGGTAGAGTCAT-3' | (Melgar-Rojas et al., 2015) |

**SUPPLEMENTRY RESULTS**

**Figure S2:** The effect of RRS-3 (3 days, 6h/day), RRS-14 (14 days, 2.5h/day) and RRS-21 (21 days, 6h/day) on body weight gain. Data expressed as group means ± SEM, n=12-16/group. *** *p*< 0.001. no RRS vs RRS.

**Figure S3:** The effect of RRS 6h/day for 3days (RRS-3) on (a) immobility time in the FST, (b) mechanical and (c) thermal heat hypersensitivity, n=6-12/group. Two-way repeated measures ANOVA followed by Newman-Keuls post-hoc: no RRS + sham vs no RRS + paw incision ^#^*p*<0.05 , ^##^*p*<0.01, ^###^*p*<0.001 , RRS + sham vs RRS + paw incision ^*^*p*<0.05 , ^**^*p*<0.01, ****p*<0.001. Data expressed as group means ± SEM.

**Figure S4:** The effect of RRS for 2.5h/day for 14 days (RRS-14) on (a) immobility time in the FST, (b) mechanical and (c) thermal heat hypersensitivity, n=8-16/group. Two-way repeated measures ANOVA followed by Newman-Keuls post-hoc: no RRS + sham vs no RRS + paw incision ^###^*p*<0.001 , RRS + sham vs RRS + paw incision ****p*<0.001. Data expressed as group means ± SEM.

**Figure S5:** The effect of RRS and/or paw incision on locomotor activity in the (a) place escape/avoidance paradigm, (b) open field test (OFT) and (c) elevated plus maze (EPM). Data expressed as group means ± SEM, n=6-8 per group.


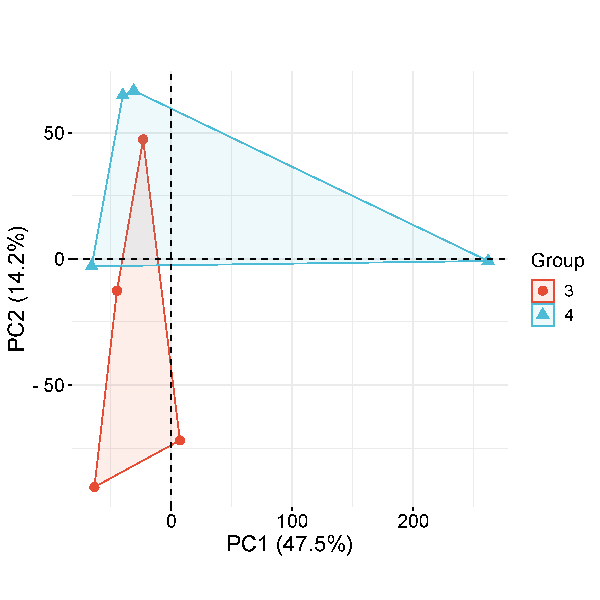


**(a)**

**(b)**


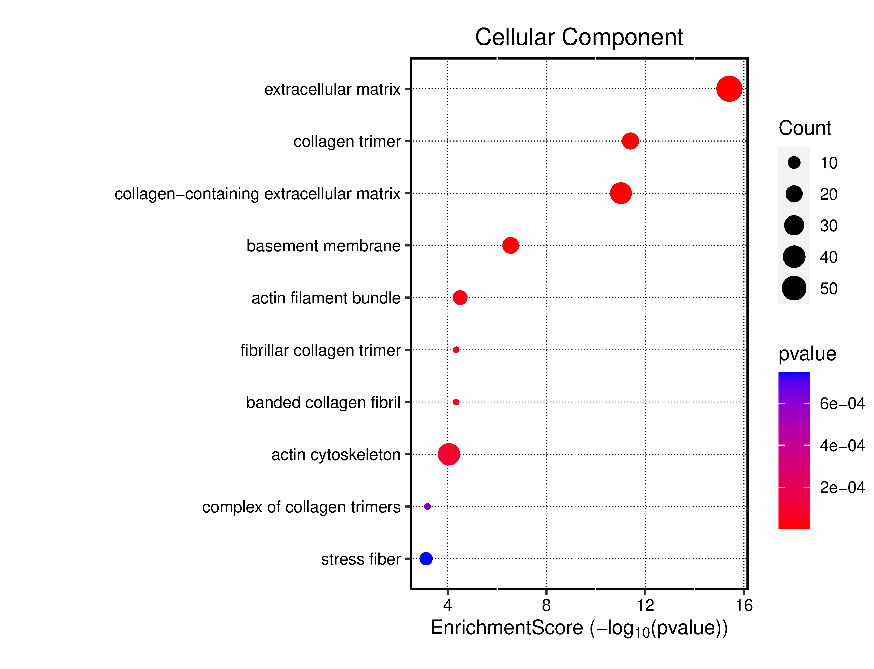


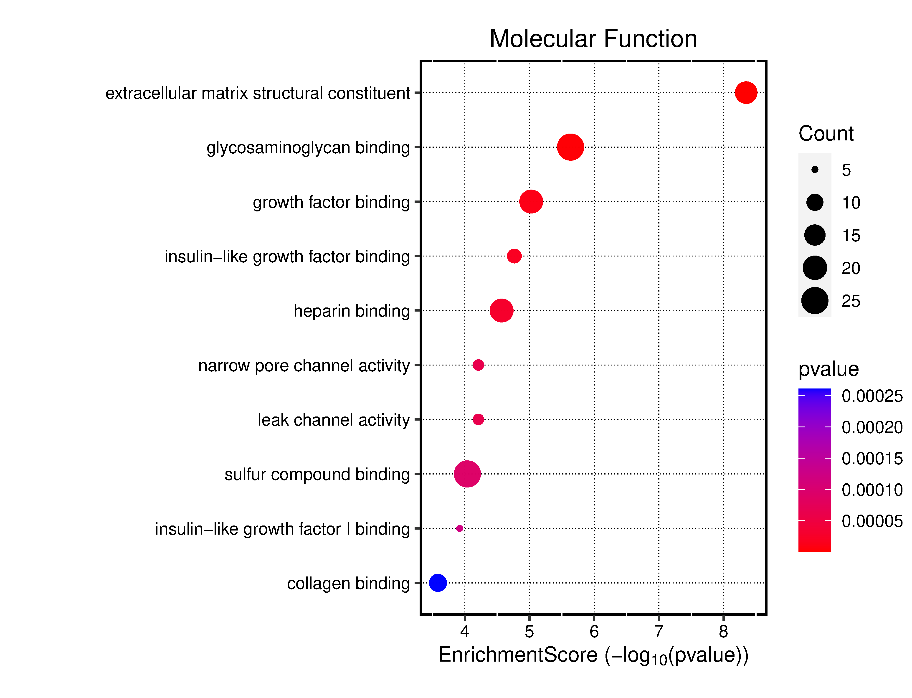


**(c)**


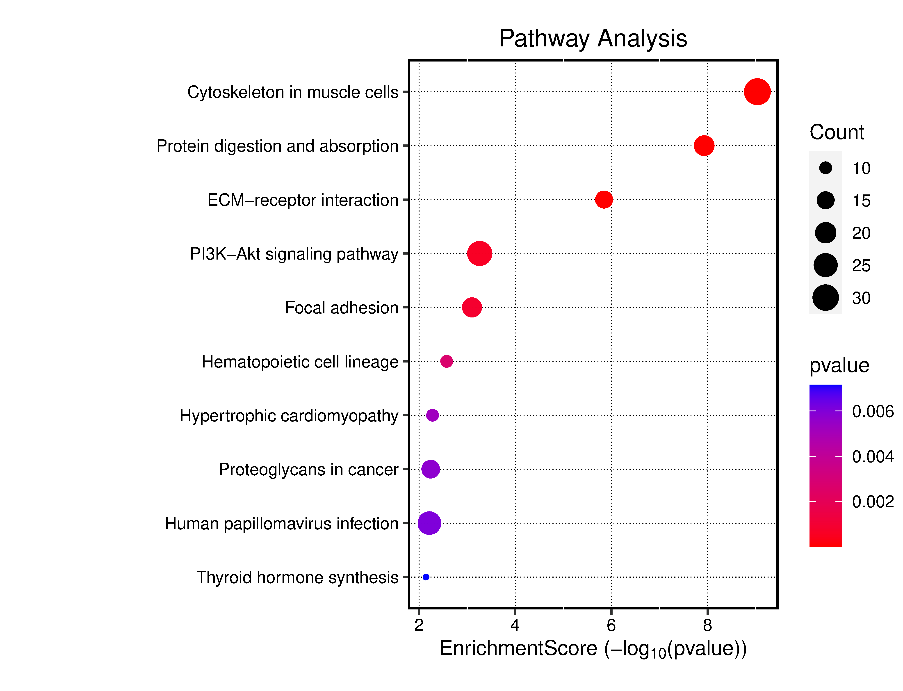


**(d)**

**(e)**

**(f)**

**Figure S6:** RNAseq analysis between no RRS + paw incision and RRS + paw incision. (a) Principal component analysis between the groups no RRS + paw incision (n=4) and RRS + paw incision (n=4). GO enrichment analysis: (b) cellular component (c) molecular function and (d) KEGG pathways of the 946 differentially expressed genes (DEG, nominally significant) in animals exposed to RRS + paw incision. (e) Paw withdrawal thresholds and (f) paw withdrawal latencies of animals used for 3’RNAseq and RT-qPCR analysis post-surgery. ^###^*p*<0.01 no RRS + sham vs no RRS + paw incision, ****p*<0.001 RRS + sham vs RRS + paw incision, ^++^*p*<0.01, ^+++^*p*<0.001. no RRS + paw incision vs RRS + paw incision. Data expressed as group means ± SEM, n=8/group.


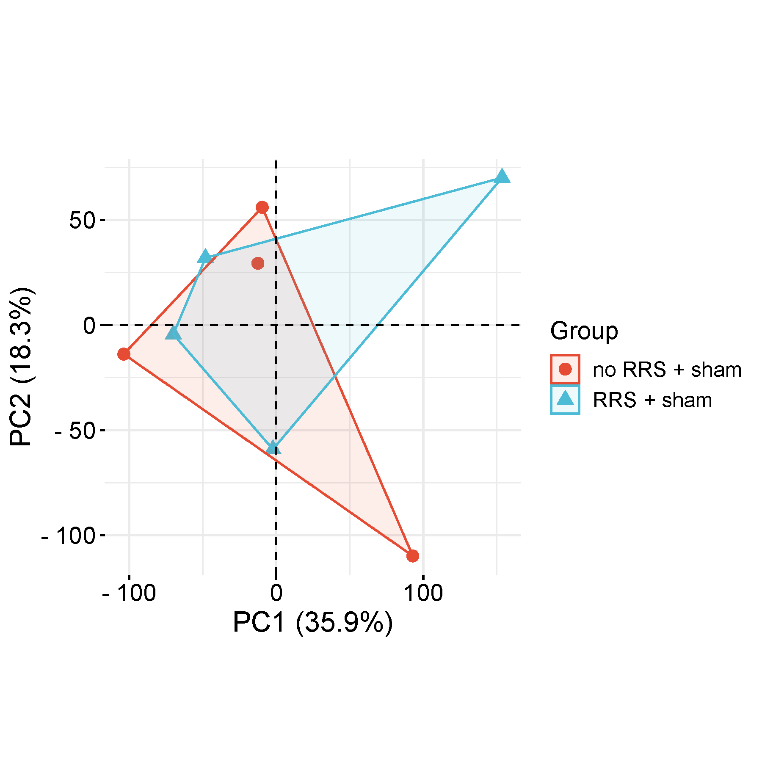
**
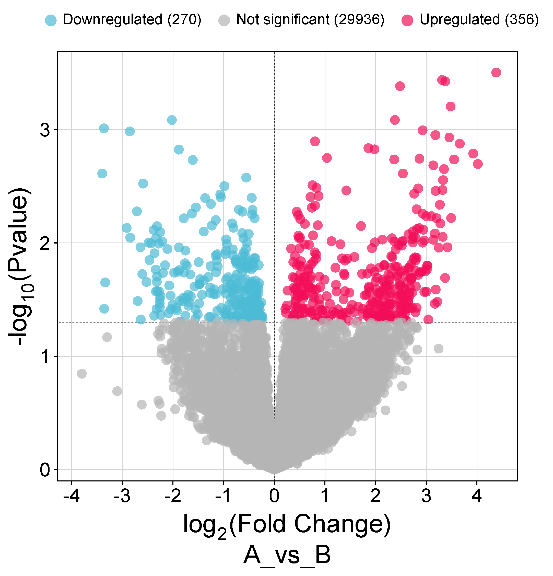
**

**(b)**

**(a)**


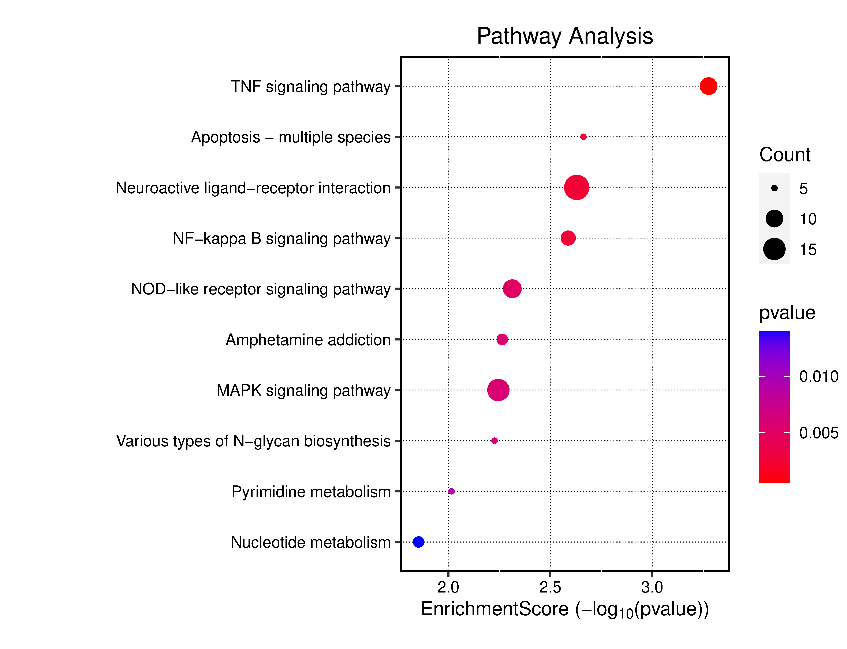

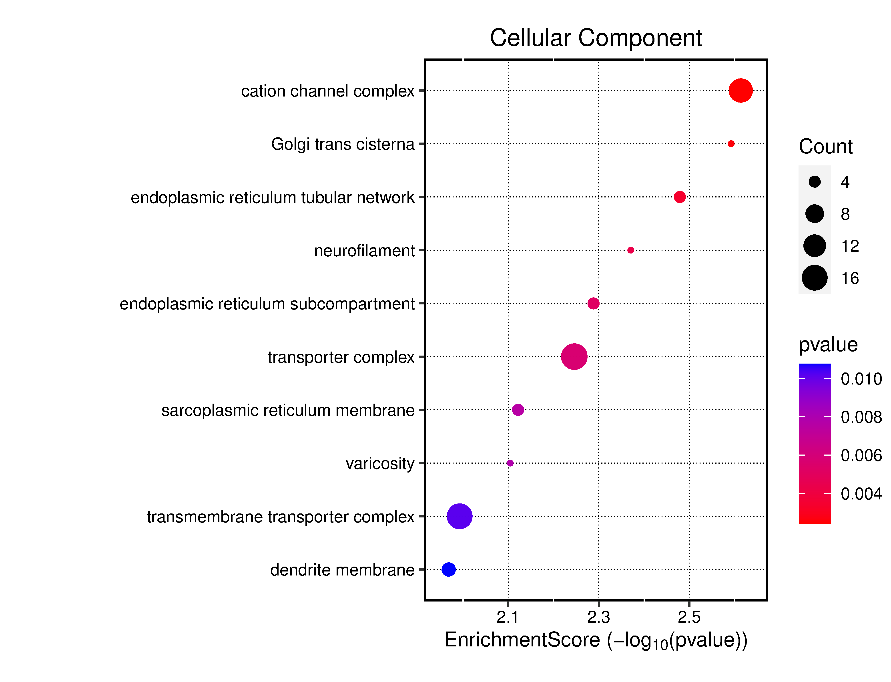
**
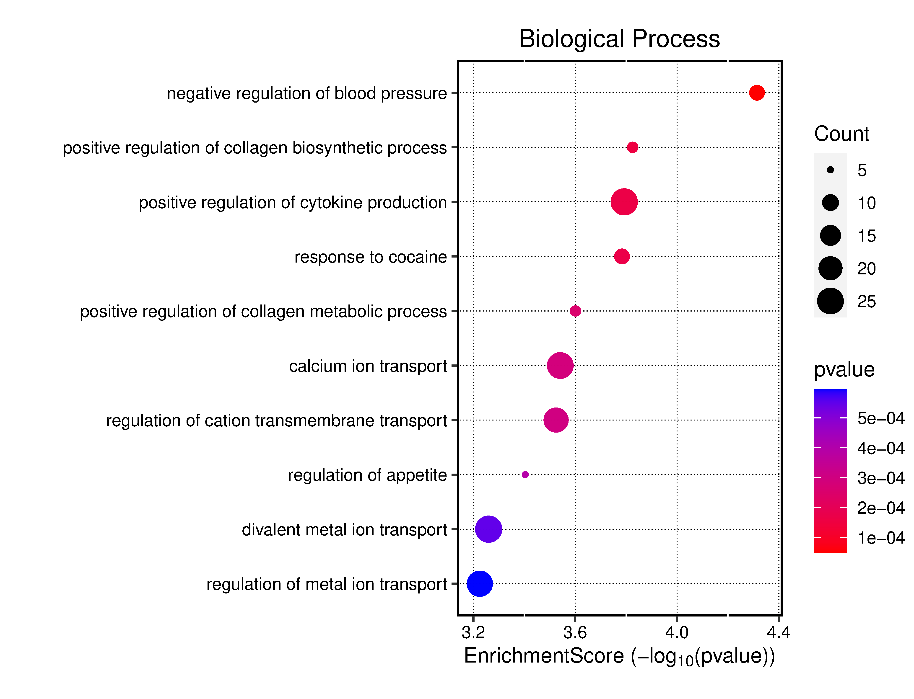

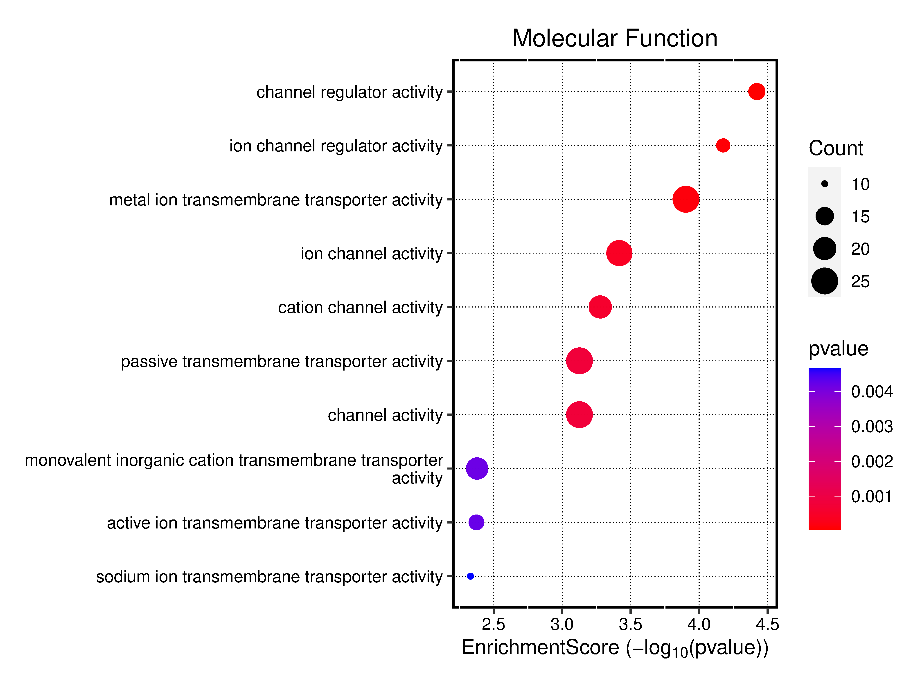
**

**(e)**

**(f)**

**(d)**

**(c)**

**Figure S7:** (a) Volcano plot depicting the 628 genes differentially expressed (nominal *P* values<0.05) between no RRS + sham (*n* = 4) and RRS + sham (*n* = 4) groups. (b) Principal component analysis between the groups no RRS + sham and RRS + sham. GO enrichment analysis: (c) biological process, (d) cellular component, (e) molecular function and (f) KEGG pathways of the differentially expressed genes (DEG, nominally significant).


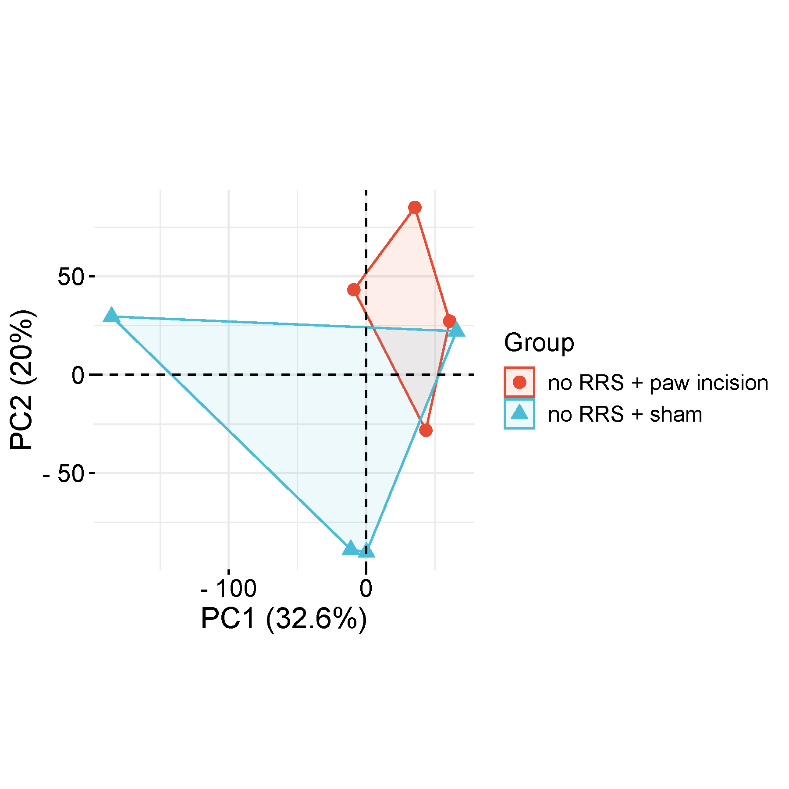

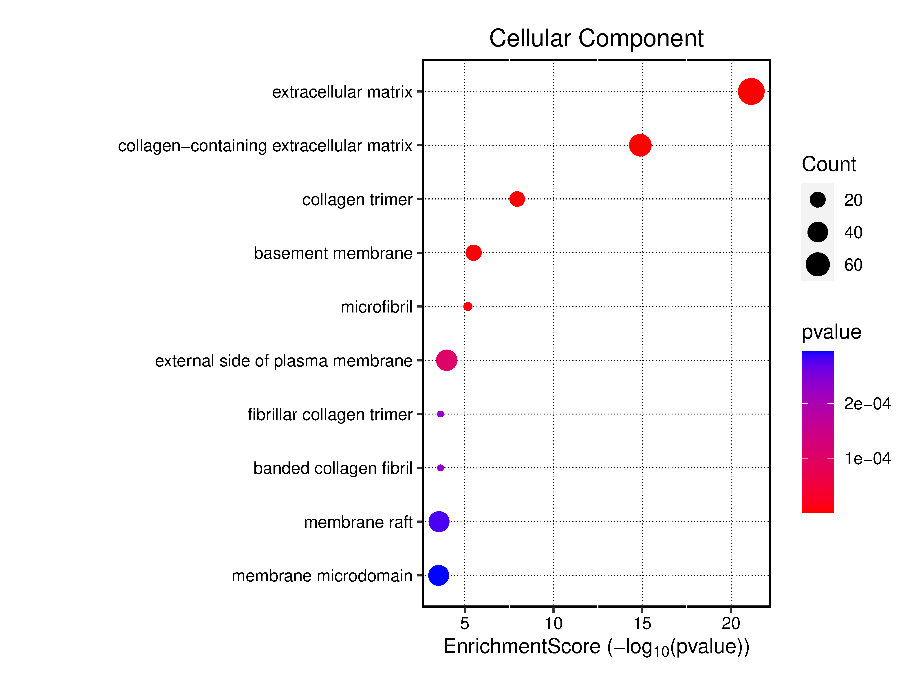

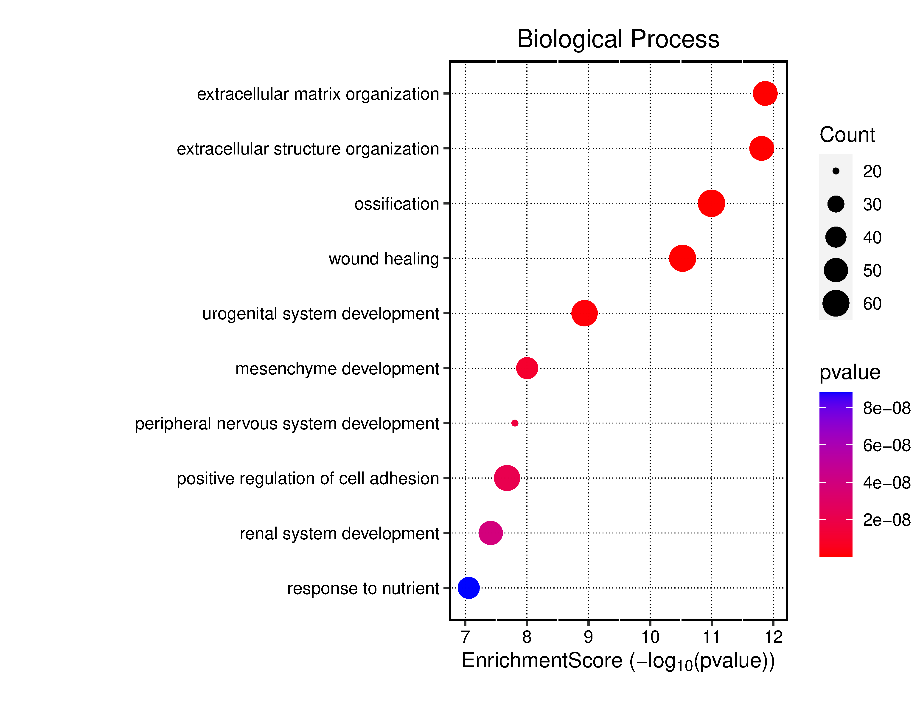

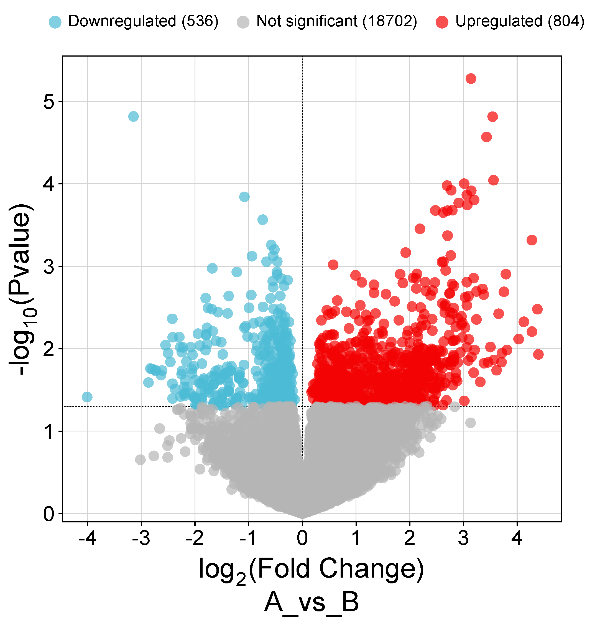

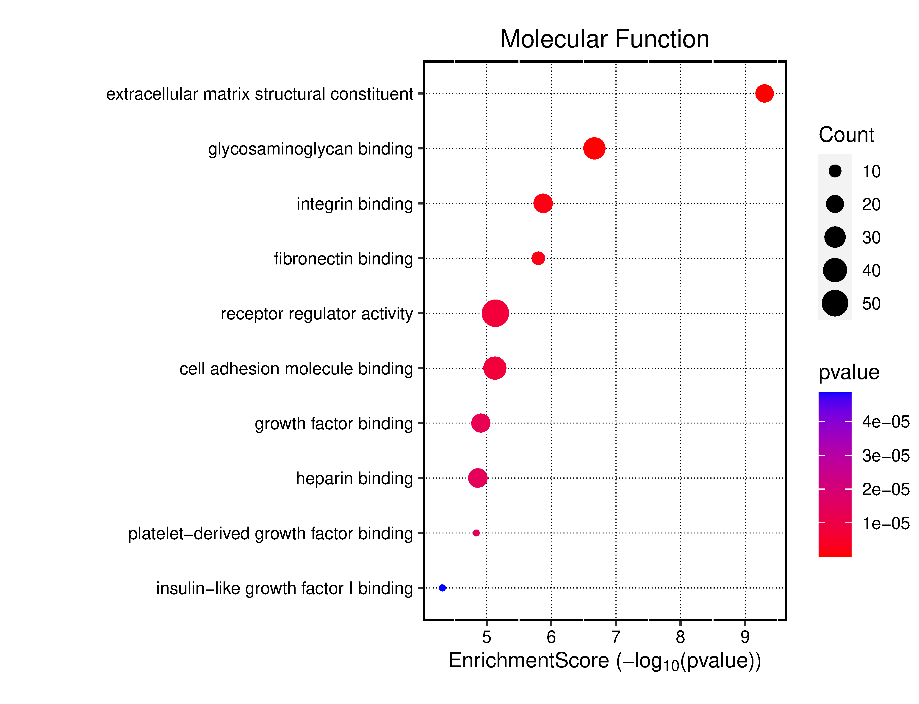

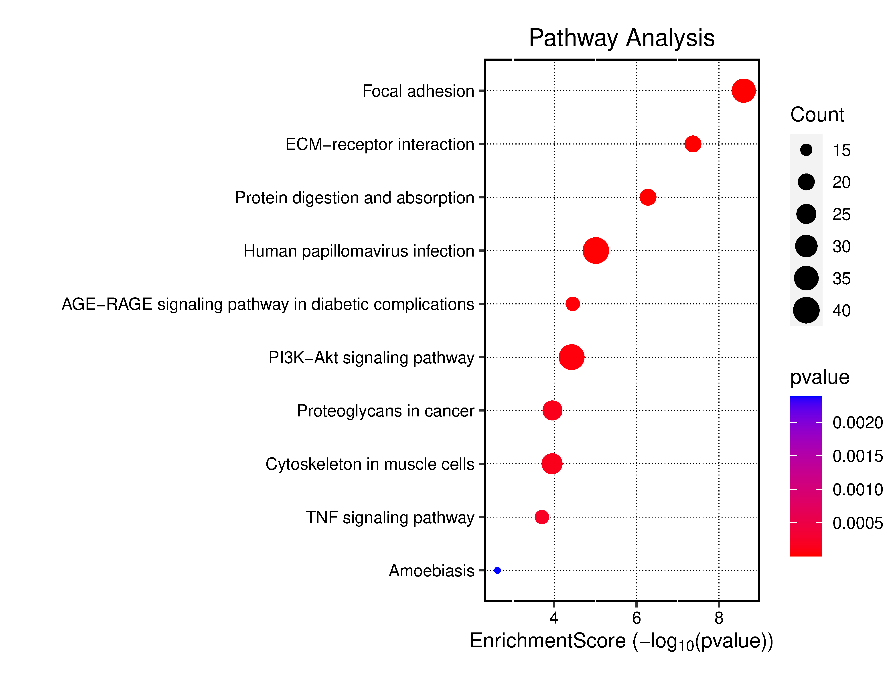


**(a)**

**(b)**

**(c)**

**(d)**

**(e)**

**(f)**

**Figure S8:** (a) Volcano plot depicting the 1340 genes differentially expressed (nominal *P* values<0.05) between no RRS + sham (*n* = 4) and no RRS + paw incision (*n* = 4) groups. (b) Principal component analysis between the groups no RRS + sham and no RRS + paw incision. GO enrichment analysis: (c) biological process, (d) cellular component, (e) molecular function and (f) KEGG pathways of the differentially expressed genes (DEG, nominally significant).


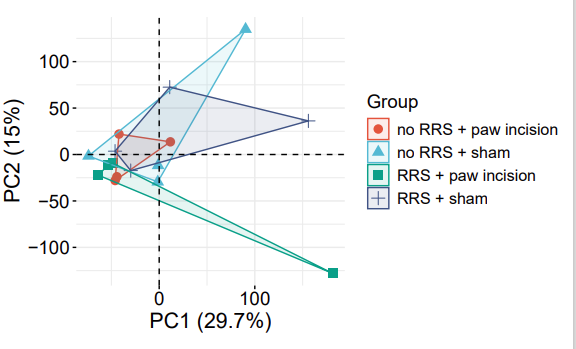


**(a)**


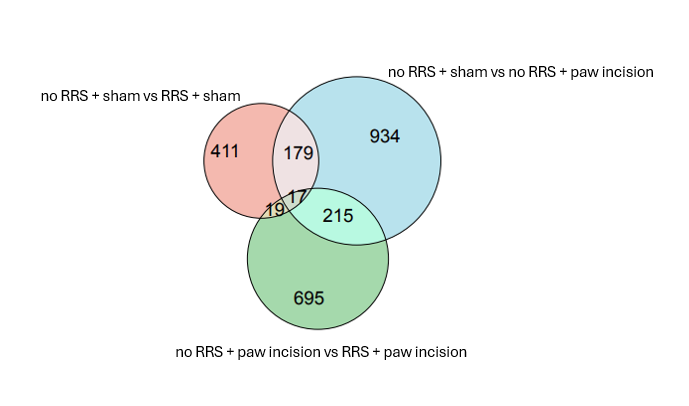


**(b)**

**Figure S9:** (a) Principal component analysis (PCA) of all four experimental groups: no RRS + sham (n = 4), RRS + sham (n = 4), no RRS + paw incision (n = 4), and RRS + paw incision (n = 4). (b) Venn diagram showing the overlap of differentially expressed genes (DEGs) among the four groups.

Foley, J. W., Zhu, C., Jolivet, P., Zhu, S. X., Lu, P., Meaney, M. J., & West, R. B. (2019). Gene expression profiling of single cells from archival tissue with laser-capture microdissection and Smart-3SEQ. *Genome Res*, *29*(11), 1816-1825. <https://doi.org/10.1101/gr.234807.118>

Galaxy, C. (2024). The Galaxy platform for accessible, reproducible, and collaborative data analyses: 2024 update. *Nucleic Acids Res*, *52*(W1), W83-W94. <https://doi.org/10.1093/nar/gkae410>

Loram, L. C., Harrison, J. A., Chao, L., Taylor, F. R., Reddy, A., Travis, C. L., Giffard, R., Al-Abed, Y., Tracey, K., Maier, S. F., & Watkins, L. R. (2010). Intrathecal injection of an alpha seven nicotinic acetylcholine receptor agonist attenuates gp120-induced mechanical allodynia and spinal pro-inflammatory cytokine profiles in rats. *Brain Behav Immun*, *24*(6), 959-967. <https://doi.org/10.1016/j.bbi.2010.03.008>

Luo, H., Xiang, Y., Qu, X., Liu, H., Liu, C., Li, G., Han, L., & Qin, X. (2019). Apelin-13 Suppresses Neuroinflammation Against Cognitive Deficit in a Streptozotocin-Induced Rat Model of Alzheimer's Disease Through Activation of BDNF-TrkB Signaling Pathway. *Front Pharmacol*, *10*, 395. <https://doi.org/10.3389/fphar.2019.00395>

Melgar-Rojas, P., Alvarado, J. C., Fuentes-Santamaria, V., Gabaldon-Ull, M. C., & Juiz, J. M. (2015). Validation of Reference Genes for RT-qPCR Analysis in Noise-Induced Hearing Loss: A Study in Wistar Rat. *PLoS One*, *10*(9), e0138027. <https://doi.org/10.1371/journal.pone.0138027>

Tang, D., Chen, M., Huang, X., Zhang, G., Zeng, L., Zhang, G., Wu, S., & Wang, Y. (2023). SRplot: A free online platform for data visualization and graphing. *PLoS One*, *18*(11), e0294236. <https://doi.org/10.1371/journal.pone.0294236>

Tomassoni, D., Avola, R., Di Tullio, M. A., Sabbatini, M., Vitaioli, L., & Amenta, F. (2004). Increased expression of glial fibrillary acidic protein in the brain of spontaneously hypertensive rats. *Clin Exp Hypertens*, *26*(4), 335-350. <https://doi.org/10.1081/ceh-120034138>

Zhang, Z., Li, Z., Ma, Z., Deng, M., Xing, M., Wu, J., Jiang, S., Wang, Q., Guo, Q., & Zou, W. (2021). Annexin A3 as a Marker Protein for Microglia in the Central Nervous System of Rats. *Neural Plast*, *2021*, 5575090. <https://doi.org/10.1155/2021/5575090>
